# Phosphatidylserine phospholipase A1 enables GPR34-dependent immune cell accumulation in the peritoneal cavity

**DOI:** 10.1084/jem.20240992

**Published:** 2024-10-16

**Authors:** Hanson Tam, Ying Xu, Jinping An, Torsten Schöneberg, Angela Schulz, Jagan R. Muppidi, Jason G. Cyster

**Affiliations:** 1 Howard Hughes Medical Institute, University of California, San Francisco, San Francisco, CA, USA; 2 Department of Microbiology and Immunology, University of California, San Francisco, San Francisco, CA, USA; 3 Medical Scientist Training Program, University of California, San Francisco, San Francisco, CA, USA; 4 Rudolf Schönheimer Institute of Biochemistry, Molecular Biochemistry, Medical Faculty, University of Leipzig, Leipzig, Germany; 5 Lymphoid Malignancies Branch, Center for Cancer Research, National Cancer Institute, National Institutes of Health, Bethesda, MD, USA

## Abstract

The peritoneal cavity (PerC) is an important site for immune responses to infection and cancer metastasis. Yet few ligand–receptor axes are known to preferentially govern immune cell accumulation in this compartment. GPR34 is a lysophosphatidylserine (lysoPS)-responsive receptor that frequently harbors gain-of-function mutations in mucosa-associated B cell lymphoma. Here, we set out to test the impact of a GPR34 knock-in (KI) allele in the B-lineage. We report that GPR34 KI promotes the PerC accumulation of plasma cells (PC) and memory B cells (MemB). These KI cells migrate robustly to lysoPS ex vivo, and the KI allele synergizes with a Bcl2 transgene to promote MemB but not PC accumulation. Gene expression and labeling studies reveal that GPR34 KI enhances PerC MemB proliferation. Both KI PC and MemB are specifically enriched at the omentum, a visceral adipose tissue containing fibroblasts that express the lysoPS-generating PLA1A enzyme. Adoptive transfer and chimera experiments revealed that KI PC and MemB maintenance in the PerC is dependent on stromal PLA1A. These findings provide in vivo evidence that PLA1A produces lysoPS that can regulate GPR34-mediated immune cell accumulation at the omentum.

## Teaser

Tam et al. report that a lymphoma-associated gain-of-function GPR34 variant promotes immune cell accumulation in the peritoneal cavity and demonstrate that this accumulation requires the lysophosphatidylserine-generating enzyme PLA1A.

## Introduction

The peritoneal cavity (PerC) encloses many abdominal organs, including the omentum, a visceral adipose tissue that contains fat-associated lymphoid clusters (FALCs) (Liu et al., 2021). Also known as milky spots, these unconventional secondary lymphoid tissues are organized by chemokines, filter PerC fluids to collect antigens and cells, and serve as hubs for local immune responses (Ansel et al., 2002; Rangel-Moreno et al., 2009; Bénézech et al., 2015). The proper recruitment, maintenance, and function of PerC immune cells are important in settings of infection (Perez-Shibayama et al., 2018; Christian et al., 2022; Yordanova et al., 2022; Newell et al., 2022) and malignancy (Flies et al., 2016; Haro et al., 2019). Immune cells are generally thought to home to the PerC through the omentum: B1 cells depend on the CXCR5–CXCL13 axis (Ansel et al., 2002), small peritoneal macrophages and inflammatory monocytes use the CCR2–CCL2 axis (Kim et al., 2016; Perez-Shibayama et al., 2018), and neutrophils are recruited by either CXCL5 (Song et al., 2013) or CXCL1 (Jackson-Jones et al., 2020). In each case, the ligand is a protein and is at least in part produced by omental stromal cells. A recent study deviated from this paradigm, showing that GPR35 on neutrophils can influence their recruitment to the inflamed PerC in response to 5-HIAA derived from platelets and mast cells (De Giovanni et al., 2022). Whether additional chemoattractant ligand–receptor pairs direct immune cell trafficking to the PerC is unknown.

GPR34 is an X-linked G protein-coupled receptor (GPCR) that is expressed in various immune cells including both myeloid and lymphoid cells (Schöneberg et al., 2018). Like many chemokine GPCRs that regulate immune cell migration and positioning, GPR34 is G_i_-coupled (Inoue et al., 2019). In vitro work has identified lysophosphatidylserine (lysoPS) as a GPR34 ligand, with the unstable *sn*-2 isomer being more potent than its *sn*-1 counterpart (Kitamura et al., 2012; Uwamizu et al., 2015). Recent cryo-EM studies have shown lysoPS positioned in the ligand-binding pocket of GPR34 in complex with the G_i_ protein (Xia et al., 2023; Liu et al., 2023; Izume et al., 2024), with docking simulations providing structural evidence that *sn*-2 is likely the physiologically bioactive form (Izume et al., 2024). The *sn*-2 isomer of lysoPS can be generated by the secreted phospholipase A1A (PLA1A) enzyme, also known as phosphatidylserine (PS)-PLA1, from PS exposed in the extracellular membrane (Makide and Aoki, 2013; Hosono et al., 2001; Izume et al., 2024). In vitro studies have shown that lysoPS stimulates various pro-growth signaling pathways (Sugo et al., 2006; Ansell et al., 2012; Korona et al., 2022) and induces cell migration (Kitamura et al., 2012) in GPR34-transfected cells.

The in vivo function of GPR34 in immune cells has received only limited study. Reports suggested that GPR34 deficiency compromises host defense against *Cryptococcus neoformans* (Liebscher et al., 2011) and reduces microglial phagocytosis (Preissler et al., 2015). Another showed that, acting in microglia, the receptor worsens neuropathic pain (Sayo et al., 2019). Moreover, it was demonstrated that apoptotic neutrophils at sites of epithelial injury release lysoPS that stimulates GPR34 on type 3 innate lymphoid cells to trigger tissue repair (Wang et al., 2021), although it was unclear how apoptotic PS was converted to lysoPS and whether the *sn*-1 or *sn*-2 isomer was involved. High tissue and serum PLA1A levels have been associated with autoimmune inflammation, cancer, and viral infection (Zhao et al., 2021), but the few studies that show in vivo PLA1A-dependent effects have not implicated GPR34 (Zhou et al., 2022; Zhao et al., 2022).

Perhaps the most prominent GPR34 connection to disease is that GPR34 translocations—associated with 10–100-fold increases in GPR34 transcript abundance—(Baens et al., 2012; Ansell et al., 2012; Akasaka et al., 2017) and putative gain-of-function (GOF) carboxy-terminal (C-terminal) mutations (Moody et al., 2018) recurrently appear in human salivary gland (SG) mucosa-associated lymphoid tissue (MALT) lymphomas. There is speculation that PLA1A expressed in autoimmune-associated SG lymphoepithelial lesions may generate lysoPS that contributes to the outgrowth of GPR34 GOF-harboring tumor cells (Korona et al., 2022). SG MALT lymphomas are neoplasms of marginal zone B cells (MZB) (Troppan et al., 2015), which are B lymphocytes named for their localization along the border of the red and the white pulp of the spleen but also found in non-lymphoid target tissues in autoimmune disease (Palm and Kleinau, 2021). Although MZB are distinct from classical memory B cells (MemB), MZB have memory properties in that they can also rapidly activate and differentiate into antibody-secreting plasma cells (PC) upon antigen encounter (Cerutti et al., 2013). While GPR34 translocations and GOF mutations are enriched in SG cases (Moody et al., 2018), GPR34 expression is generally increased in MALT, MZB, and lymphoplasmacytic lymphomas (Ansell et al., 2012), suggesting a broader pathological relevance of the receptor.

In an effort to model human lymphoma, we generated B cell–specific GPR34 GOF knock-in (KI) mice. Our analyses of these mice revealed an unexpected accumulation of PC and MemB in the PerC. We report that ex vivo PerC KI cells strongly migrate to lysoPS and that GPR34 KI augments PerC MemB proliferation. PLA1A was found to produce *sn*-2 lysoPS at the omentum, where KI PC and MemB are enriched. We show that stromal-derived enzyme is required for the GPR34-dependent maintenance of KI cells in the PerC.

## Results and discussion

### Generation of GPR34 GOF KI mice

**Figure S1.**
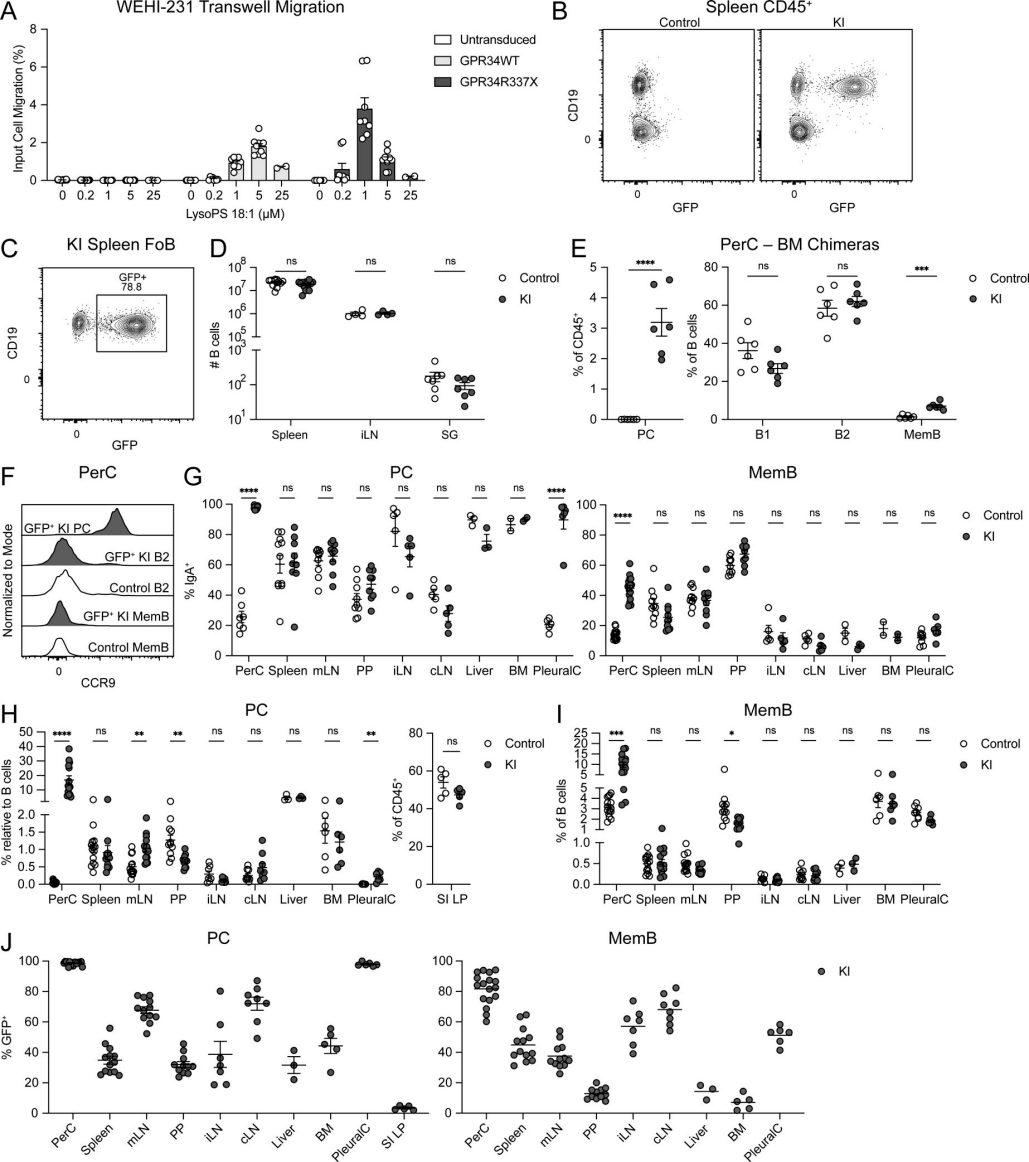
**Characterization of GPR34 KI mice. (A)** WT or R337X mouse GPR34 was overexpressed in WEHI-231 B lymphoma cells by retroviral transduction. Cells were assayed for transwell migration to the indicated concentrations of lysoPS 18:1. Bar graph shows the percentage of migrated cells relative to input. **(B–J)** Immune cells from control and KI (*Cd21*^*Cre*^*R26*^*LSL-Gpr34Q340X-IRES-GFP*^) mice were analyzed by flow cytometry. **(B)** Representative flow cytometry plots of spleen CD45^+^ cells showing GFP reporter expression in KI B cells (CD19^+^). **(C)** Representative flow cytometry plot of KI spleen FoB gated for GFP reporter expression and annotated with GFP^+^ percentage. **(D)** Cell numbers of B cells from the spleen, iLN, and SG of control and KI mice (spleen, *n* = 12; iLN, *n* = 4; SG, *n* = 7). **(E)** Frequencies of PerC PC (CD138^+^), B1 (IgD^−^IgM^+^), B2 (IgD^+^IgM^+^), and MemB (IgD^−^IgM^−^) in chimeric mice with control or KI BM reconstituted into WT hosts (control, *n* = 6; KI, *n* = 6). **(F)** Representative flow cytometry histograms of CCR9 expression in the indicated PerC populations. **(G–J)** Data for iLN, cLN, and liver each include three BM chimeric mice. PC were gated as B220^−^IgA^+^ in the SI LP and CD138^+^ otherwise. MemB were gated as IgD^−^IgM^−^ in the PerC, IgD^−^IgM^−^CD38^+^GL7^−^ in the BM, and IgD^−^CD38^+^GL7^−^CD95^+^ otherwise. **(G)** Frequencies of IgA^+^ cells within PC and MemB in the indicated organs (control PerC PC, *n* = 7; other PerC, *n* = 16–17; spleen, *n* = 9–11; mLN, *n* = 9–10; PP, *n* = 9; iLN, *n* = 5; cLN, *n* = 5; liver, *n* = 3; BM, *n* = 2; PleuralC, *n* = 5–8). **(H and I)** Frequencies of PC and MemB in the indicated organs (PerC, *n* = 14–15; spleen, *n* = 14; mLN, *n* = 13–14; PP, *n* = 11; iLN, *n* = 7; cLN, *n* = 9; liver, *n* = 3; BM, *n* = 6; PleuralC, *n* = 6–8; SI LP, *n* = 5). **(J)** Frequencies of GFP^+^ cells within PC and MemB in the indicated organs (PerC, *n* = 17; spleen, *n* = 13; mLN, *n* = 12; PP, *n* = 11; iLN, *n* = 7; cLN, *n* = 8; liver, *n* = 3; BM, *n* = 5; PleuralC, *n* = 6, SI LP, *n* = 5). Each data point represents an individual transwell (A) or mouse (D, E, and G–J), lines indicate means, and error bars represent SEM. Data were pooled from three or more independent experiments, except in D and G, which were pooled from two or more independent experiments. Statistical significance (D, E, and G–J) was determined by unpaired *t* test corrected for multiple comparisons (Holm-Šídák). ns, not significant; *P < 0.05; **P < 0.01; ***P < 0.001; ****P < 0.0001.

**Figure 1.**
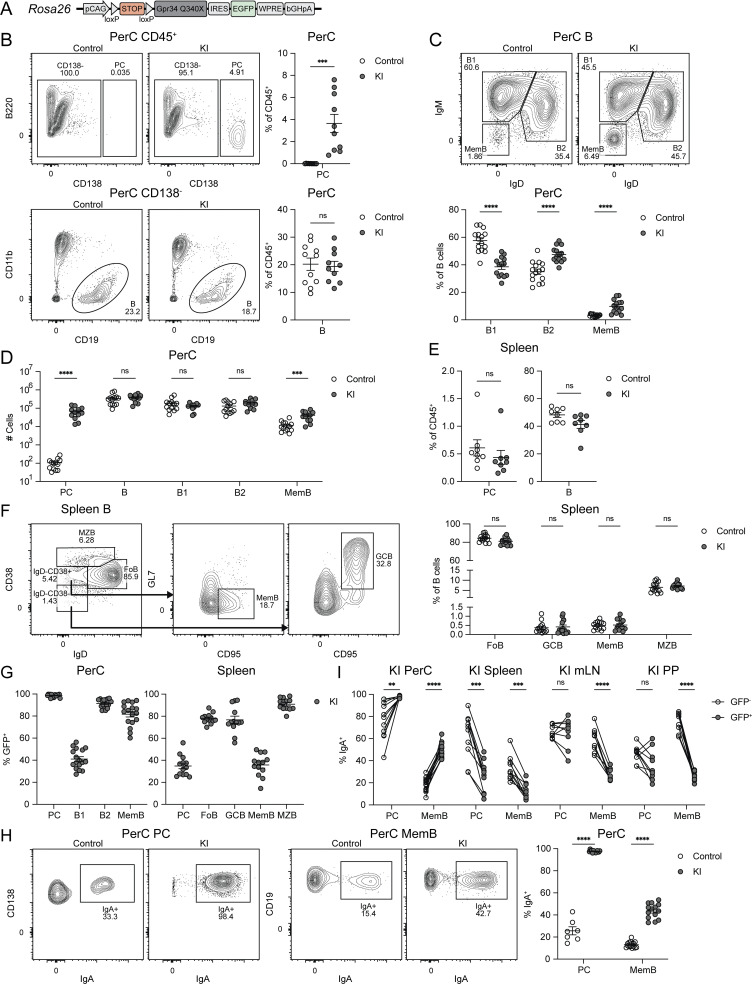
**GPR34 KI promotes PC and MemB accumulation in the PerC. (A)** Diagram of the GPR34 GOF conditional KI allele situated at the *Rosa26* locus. **(B–I)** Immune cells from control and KI (*Cd21*^*Cre*^*R26*^*LSL-Gpr34Q340X-IRES-GFP*^) mice were analyzed by flow cytometry. **(B)** Left top: Representative flow cytometry plots of PerC CD45^+^ cells gated for PC (CD138^+^) and annotated with PC frequency. Left bottom: Representative flow cytometry plots of PerC CD45^+^CD138^−^ cells gated for B cells (CD19^+^) and annotated with B cell frequency. Right: Percentages of PerC PC and B cells amongst CD45^+^ cells (control, *n* = 11; KI, *n* = 10). **(C)** Top: Representative flow cytometry plots of PerC B cells gated for B1 (IgD^−^IgM^+^), B2 (IgD^+^IgM^+^), and MemB (IgD^−^IgM^−^) and annotated with subset frequencies. Bottom: Percentages of PerC B1, B2, and MemB amongst B cells (control, *n* = 15; KI, *n* = 14). **(D)** Cell numbers of the indicated populations in the PerC (control, *n* = 13; KI, *n* = 12). **(E)** Frequencies of the indicated populations in the spleen (control, *n* = 8; KI, *n* = 8). **(F)** Left: Representative flow cytometry plots showing the gating scheme for spleen FoB (IgD^+^CD38^+^), GCB (IgD^−^CD38^−^GL7^+^CD95^+^), MemB (IgD^−^CD38^+^GL7^−^CD95^+^), and MZB (IgD^int^CD38^hi^). Right: Percentages of FoB, GCB, MemB, and MZB amongst spleen B cells (control, *n* = 14; KI, *n* = 14). **(G)** Frequencies of GFP^+^ cells within the indicated populations in the PerC and spleen (PerC, *n* = 17; spleen, *n* = 13). **(H)** Left: Representative flow cytometry plots of PerC PC and MemB gated for IgA^+^ cells and annotated with IgA^+^ frequency. Right: Percentages of IgA^+^ cells within the indicated populations in the PerC (control PC, *n* = 7; control MemB, *n* = 15; KI, *n* = 14). **(I)** Frequencies of IgA^+^ cells within the GFP^−^ and GFP^+^ subsets of the indicated populations in KI mice (PerC PC, *n* = 11; PerC MemB, *n* = 15; spleen, *n* = 10; mLN, *n* = 9; PP, *n* = 9). In B–H, each data point represents an individual mouse, lines indicate means, and error bars represent SEM. In I, each pair of connected points represents data from one mouse. Data were pooled from three or more independent experiments. Statistical significance was determined by unpaired *t* test (B–F and H) or paired *t* test (I) corrected for multiple comparisons (Holm-Šídák). ns, not significant; **P < 0.01; ***P < 0.001; ****P < 0.0001.

Truncations are common among the C-terminal GPR34 mutations in SG MALT lymphoma (Moody et al., 2018). Of note, similar mutations in other GPCRs, in particular CXCR4 and CCR4, are GOF and can lead to malignant transformation (Martinez-Climent, 2018). To test the functional effect of GPR34 truncations, we retrovirally transduced either mouse R337X or wild-type (WT) GPR34 into the WEHI-231 mouse B lymphoma cell line. In accord with a report using transfected epithelial cells (Kitamura et al., 2012), overexpression of WT GPR34 enabled concentration-dependent transwell migration of WEHI-231 cells to lysoPS (Fig. S1 A). Meanwhile, R337X exhibited GOF behavior with a larger and more sensitive response (Fig. S1 A) in line with the enhanced signaling and growth properties of a different C-terminal truncation (Korona et al., 2022). Attempts to study both the WT and GOF versions of the receptor in vivo through retroviral overexpression bone marrow (BM) chimeras were limited by plasmid toxicity effects that made the GPR34 construct unstable and prone to mutation during growth in bacteria (unpublished data). As an alternative approach, we generated mice harboring a conditional GPR34 GOF KI allele with a GFP reporter (Fig. 1 A). For this model, we chose mouse Q340X, which is homologous to human Q347X, the most frequent GPR34 mutation found in SG MALT lymphoma (Moody et al., 2018). In *Cd21*^*Cre*^*R26*^*LSL-Gpr34Q340X-IRES-GFP*^—henceforth “KI”—mice, reporter expression in the hematopoietic compartment was restricted to B-lineage cells (Fig. S1 B). Consistent with previous studies of *Cd21*^*Cre*^-mediated recombination efficiency (Kraus et al., 2004), the KI allele was expressed in ∼80% of splenic follicular B cells (FoB) (Fig. S1 C).

### GPR34 KI promotes the PerC accumulation of PC and MemB

Flow cytometric profiling of KI mice at homeostasis revealed normal numbers of B cells in the spleen and inguinal lymph nodes (iLN) (Fig. S1 D). Analysis of the SG identified very few B cells in control mice and no accumulation in the GPR34 GOF setting (Fig. S1 D). Profiling of B-lineage cells in additional tissues revealed a marked accumulation of KI PC in the PerC. While CD138^+^ PC were virtually absent from the PerC of controls, they constituted ∼3% of PerC immune cells in KI mice (Fig. 1 B). Within B cells, we observed a relative decrease in B1 (IgD^−^IgM^+^) and a relative increase in B2 (IgD^+^IgM^+^) and class-switched MemB (IgD^−^IgM^−^), but the overall B cell frequency was unaffected (Fig. 1, B and C). Numerically, there was a ∼500-fold increase in PC and an approximately threefold increase in MemB (Fig. 1 D). Outside of the B-lineage, *Cd21*^*Cre*^ is active in stromal follicular dendritic cells. We reconstituted WT mice with BM from KI donors and found similar PerC accumulation of PC and MemB (Fig. S1 E), indicating that the observed phenotypes are B cell intrinsic.

In contrast to the PerC, PC and MemB were not expanded in the spleen (Fig. 1, E and F). Splenic FoB, germinal center B cells (GCB), and MZB were also unaffected (Fig. 1 F). We observed lower KI GFP reporter positivity for splenic PC and MemB relative to their counterparts in the PerC (Fig. 1 G). To a lesser extent, there was also lower KI GFP^+^ frequency amongst splenic FoB compared with PerC B2 (Fig. 1 G). The high GFP reporter positivity in splenic MZB (Fig. 1 G) is in accord with high *Cd21*^*Cre*^ activity in these CD21^hi^ cells. The enrichment of GFP^+^ PC, MemB, and B2 in the PerC compared with the spleen is consistent with the GPR34 GOF allele promoting accumulation of these cell types in the PerC.

We found that PerC KI PC were nearly 100% IgA^+^ (Fig. 1 H) and expressed CCR9 (Fig. S1 F), which are properties of gut-associated PC (Pabst et al., 2004). PerC KI MemB also had increased IgA positivity (Fig. 1 H), and within KI mice, GFP^+^ PerC PC and MemB had higher IgA^+^ percentages than their GFP^−^ counterparts (Fig. 1 I). With one exception, no significant IgA^+^ frequency differences were identified in other surveyed organs at the gross population level (Fig. S1 G). These data make it unlikely that the GPR34 GOF allele is directly promoting IgA class switching. Subsetting by GFP positivity revealed lower IgA^+^ proportions within reporter-positive spleen PC and MemB as well as MemB in the mesenteric LN (mLN) and the Peyer’s patches (PP) (Fig. 1 I). Additionally, we observed lower frequencies and low GFP^+^ percentages of KI PC and MemB in the PP (Fig. S1, H–J).

While control IgA^+^ PC home via the circulation preferentially to the small intestine (SI) lamina propria (LP) (Isho et al., 2021), their KI counterparts may instead be diverted to the PerC. This aligns with the observation that only ∼5% of KI SI LP PC were GFP^+^ compared with ∼30% in the PP (Fig. S1 J), revealing a strong bias against KI PC accumulation in the gut LP. SI LP PC abundance did not significantly differ between KI and control (Fig. S1 H), indicating that reporter-negative IgA^+^ PC fill the niche. Comparison across organs highlights the PerC as the site with the largest PC and MemB frequency differences (Fig. S1, H and I) and the strongest enrichment for KI reporter expression (Fig. S1 J), consistent with the PerC being a destination of IgA^+^ KI PC and MemB. The pleural cavity (PleuralC) shares certain immunological properties with the PerC (Ansel et al., 2002), and we observed that KI PC but not MemB had increased frequency, increased IgA positivity, and high GFP reporter positivity at this site (Fig. S1, G–J).

Although the mLN showed a reduction in IgA^+^ MemB amongst the GFP^+^ KI population (Fig. 1 I), there was an increased frequency and high GFP positivity of KI PC in this tissue (Fig. S1, H and J). High GFP positivity was also observed amongst PCs and MemB in the cervical LN (cLN) (Fig. S1 J). These data suggest there may be additional effects of the KI allele beyond those on IgA^+^ cells that influence cell migration or persistence in lymphoid tissues.

### PC and B cell abundance are unaltered in GPR34-deficient mice

**Figure S2.**
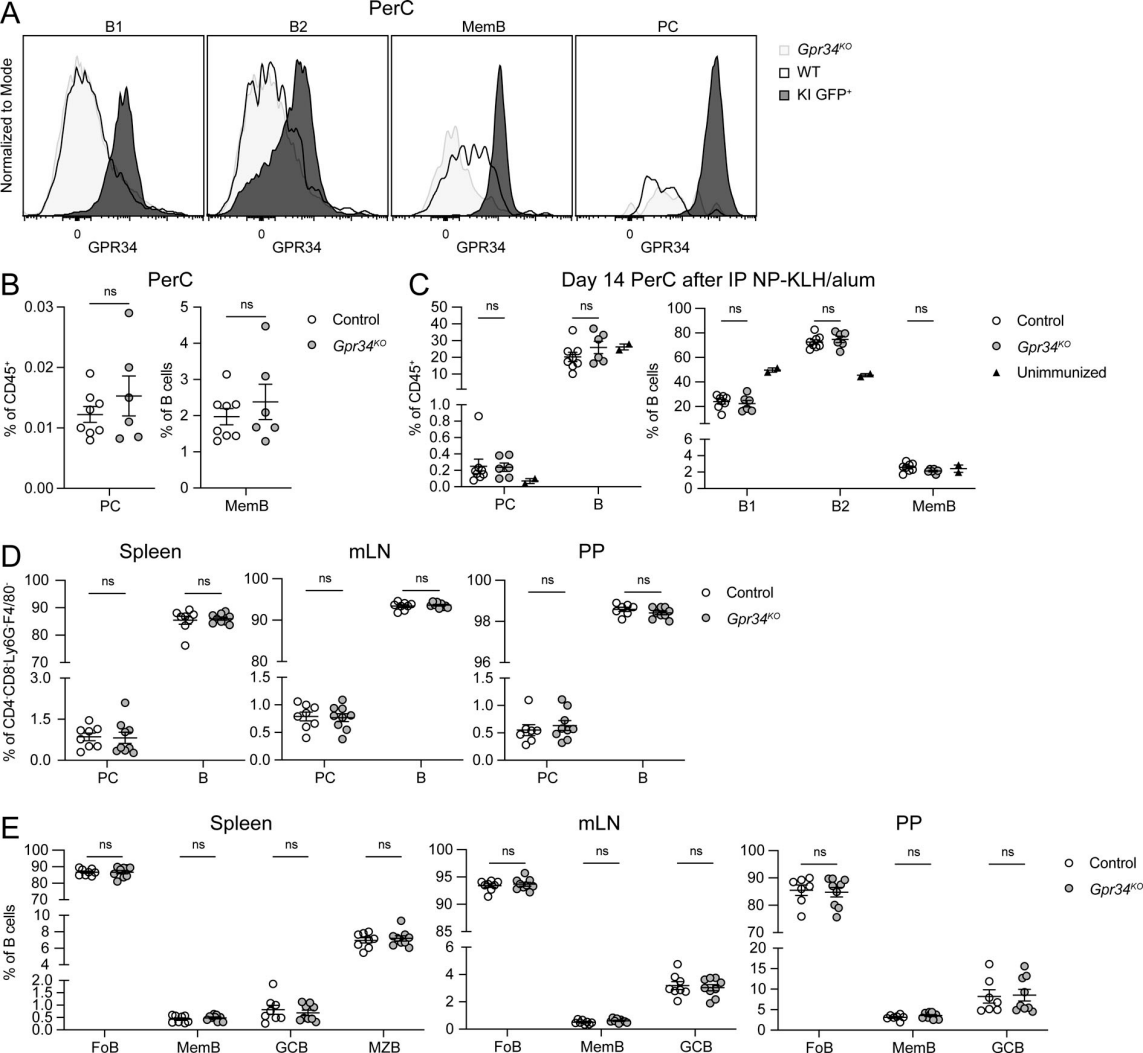
**GPR34 deficiency does not result in PC or B cell accumulation defects. (A)** Representative flow cytometry histograms of polyclonal anti-GPR34 antibody surface staining of the indicated PerC populations from *Gpr34*^*KO*^, WT, and KI (*Cd21*^*Cre*^*R26*^*LSL-Gpr34Q340X-IRES-GFP*^) mice. **(B–E)** Immune cells from control and *Gpr34*^*KO*^ mice were analyzed by flow cytometry. **(B)** Frequencies of PC (CD138^+^) and MemB (IgD^−^IgM^−^) in the PerC (control, *n* = 8; *Gpr34*^*KO*^, *n* = 6). **(C)** Frequencies of PC, B, B1 (IgD^−^IgM^+^), B2 (IgD^+^IgM^+^), and MemB in the PerC 14 days after mice were immunized IP with NP-KLH in alum adjuvant or left unimmunized (control, *n* = 8; *Gpr34*^*KO*^, *n* = 6; unimmunized, *n* = 2). **(D and E)** Frequencies of PC, B, FoB (IgD^+^CD38^+^), MemB (IgD^−^CD38^+^GL7^−^CD95^+^), GCB (IgD^−^CD38^−^GL7^+^CD95^+^), and MZB (IgD^int^CD38^hi^) in the indicated organs (control, *n* = 7–8; *Gpr34*^*KO*^, *n* = 9). Each data point represents an individual mouse (B–E), lines indicate means, and error bars represent SEM. Data were pooled from two independent experiments. Statistical significance (B–E) was determined by unpaired *t* test corrected for multiple comparisons (Holm-Šídák). ns, not significant.

Given the phenotypes observed with GPR34 GOF overexpression, we wondered if GPR34 deficiency would result in defects in PC or MemB populations. Surface staining for GPR34 using a polyclonal antibody demonstrated that WT PerC MemB expressed a low level of the receptor (Fig. S2 A), consistent with transcriptional data for MemB in the spleen (Bhattacharya et al., 2007; Duan et al., 2021). GPR34 protein was not detected on WT B1, B2, or PC (Fig. S2 A). As expected, cells from KI mice exhibited strong GPR34 protein expression (Fig. S2 A). At homeostasis, there was no reduction of PerC PC and MemB frequencies in GPR34-deficient mice (Fig. S2 B). Intraperitoneal (IP) immunization with nitrophenyl-haptenated keyhole limpet hemocyanin (NP-KLH) in alum adjuvant induced an overall increase in PerC PC at 2 wk but did not reveal any GPR34-dependent changes in PC or B cell frequency (Fig. S2 C). PC and B cell abundance also did not differ in the spleen, mLN, and PP at homeostasis (Fig. S2, D and E). These results indicate that endogenous levels of GPR34 on MemB are insufficient to alter B cell population frequencies in the contexts we studied.

### GPR34 KI cells migrate to lysoPS ex vivo

**Figure 2.**
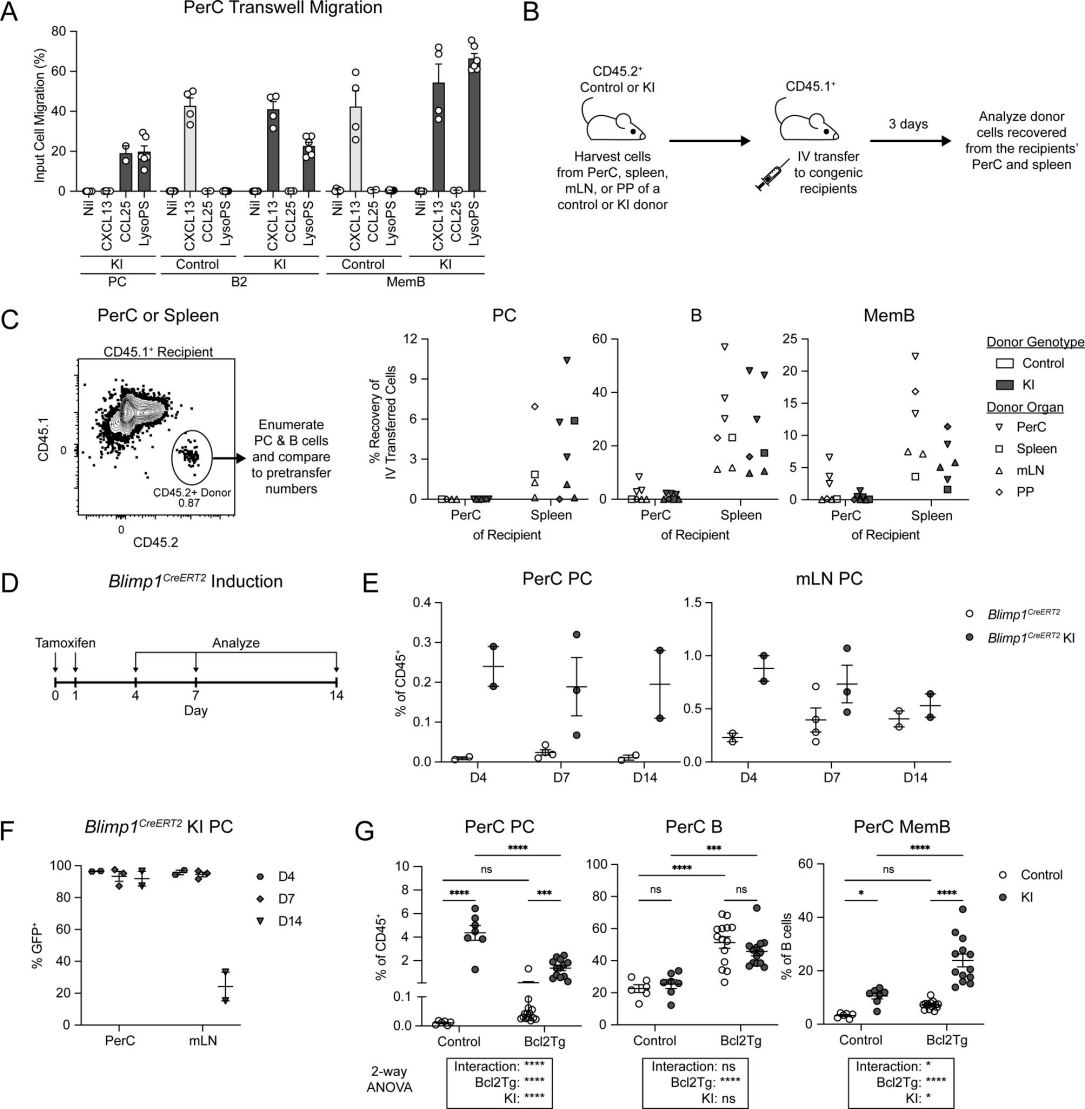
**GPR34 KI supports ex vivo lysoPS-mediated chemotaxis, can conditionally promote PerC PC accumulation, and synergizes with BCL2 to enhance PerC MemB abundance. (A)** PerC cells were harvested from control and KI (*Cd21*^*Cre*^*R26*^*LSL-Gpr34Q340X-IRES-GFP*^) mice and assayed for transwell migration to 1 µg/ml CXCL13, 5 µg/ml CCL25, or 5 µM lysoPS 18:1. Bar graph shows the frequency of PC (CD138^+^), B2 (IgD^+^IgM^+^), and MemB (IgD^−^IgM^−^) migration relative to input (nil, *n* = 6; CXCL13, *n* = 4; CCL25, *n* = 2; LysoPS, *n* = 6). **(B–G)** Immune cells were analyzed by flow cytometry. **(B and C)** PerC, spleen, mLN, and PP cells were harvested from control and KI mice, followed by adoptive transfer IV into congenic recipients. 3 days later, recipients’ PerC and spleen were analyzed for donor cells. **(B)** Schematic of IV adoptive transfer experiment. **(C)** Left: Representative flow cytometry plot identifying CD45.2^+^ congenic donor cells in samples harvested from CD45.1^+^ recipients. Right: Plots show the percent of pretransfer donor cells recovered from the recipients’ PerC and spleen. PerC donor MemB were gated as IgD^−^IgM^−^ and donor MemB from other organs were gated as IgD^−^CD38^+^ (control, *n* = 6–7; KI, *n* = 6–7; donor control PerC PC abundance was insufficient for quantification of their recovery from recipients). **(D–F)** *Blimp1*^*CreERT2*^ and *Blimp1*^*CreERT2*^ KI (*Blimp1*^*CreERT2*^*R26*^*LSL-GPR34Q340X-IRES-GFP*^) BM chimeric mice received tamoxifen via oral gavage on days 0 and 1 and were analyzed on days 4, 7, or 14 (D4, *n* = 2; D7, *n* = 3–4; D14, *n* = 2). **(D)** Timeline of tamoxifen induction and analysis. **(E)** Frequency of PerC and mLN PC at the indicated timepoints. **(F)** Frequency of GFP^+^ cells within PerC and mLN PC at the indicated timepoints. **(G)** Frequency of the indicated PerC populations in control and KI mice with or without the E*µ-Bcl2* transgene (control, *n* = 6; KI, *n* = 7; Bcl2Tg, *n* = 14; Bcl2Tg KI, *n* = 13). Each data point represents an individual transwell (A) or mouse (C and E–G), lines indicate means, and error bars represent SEM. Data were pooled from three independent experiments, except in E and F, which were pooled from two experiments. Statistical significance (G) was determined by two-way ANOVA, followed by multiple comparisons using Fisher’s LSD test. ns, not significant; *P < 0.05; ***P < 0.001; ****P < 0.0001.

To investigate whether chemotaxis may contribute to the accumulation of KI cells in the PerC, we first evaluated their transwell migration to various chemoattractants. Both control and KI B2 and MemB responded to CXCL13, but only the KI populations migrated to lysoPS (Fig. 2 A). KI PC also showed migration toward the CCR9 ligand CCL25 (Fig. 2 A), agreeing with CCR9 expression levels (Fig. S1 F). We next tested whether intravenously (IV) transferred KI cells could home to the PerC (Fig. 2 B). Regardless of their genotype or anatomical origin, at 3 days after IV transfer, donor B cells, PC, and MemB were less recovered from the PerC than from the spleen; in particular, donor KI PC were not detected in the PerC of recipients, and donor KI MemB showed no homing advantage over their control counterparts (Fig. 2 C). Our inability to observe KI PC trafficking from the blood into the PerC may be explained by limited donor cell numbers and altered properties of ex vivo–manipulated cells. Inefficient entry into the PerC in short-term homing assays (Fig. 2, B and C) might be counteracted in KI mice by a continuous flux of KI cells through circulation. It is also possible that KI PC and MemB arrive in the PerC via a non-hematogenous route or that they develop locally in the PerC.

### Conditional activation of GPR34 KI is sufficient for PerC PC accumulation

Both PC and MemB are prominently affected in the *Cd21*^*Cre*^ model, which promotes the expression of the GOF receptor at the mature B cell stage. To assess the effect of expressing GPR34 KI specifically at the PC stage, we bred the KI allele with *Blimp1*^*CreERT2*^ mice that also have a tdTomato reporter at the *Blimp1* locus (Robinson et al., 2023). 8 wk after reconstitution, *Blimp1*^*CreERT2*^*R26*^*LSL-GPR34Q340X-IRES-GFP*^ BM chimeric mice—“Blimp1Cre KI”—received tamoxifen via oral gavage on days 0 and 1, followed by analysis at various timepoints (Fig. 2 D). By day 4, Blimp1Cre KI PC were strongly accumulated in the PerC, though at a frequency ∼10-fold lower than in the *Cd21*^*Cre*^ model; this level persisted at least through day 14 (Fig. 2 E). An increase in mLN PC was also present at day 4 but subsequently waned (Fig. 2 E). These data correlated with the maintenance of high Blimp1Cre KI PC GFP reporter positivity in the PerC even after it dropped in the mLN pursuant to tamoxifen clearance (Fig. 2 F). Thus, GPR34 KI can conditionally promote PerC PC accumulation when turned on at the PC stage, and KI PC selectively persist in the PerC after seeding.

### KI PerC MemB accumulation is enhanced by Bcl2 overexpression

To explore the interaction of cell survival signals with the maintenance of PerC PC and MemB, we used Eµ*-bcl2* mice (Strasser et al., 1991), in which anti-apoptotic Bcl2 protein is expressed in the B-lineage, leading to overall expansion of B cells, including the splenic PC and MemB compartments (Smith et al., 1994, 2000). In the PerC, there were trends for increases in both PC and MemB in Bcl2 transgenic compared with control mice (Fig. 2 G). On a GPR34 KI background, the addition of the Bcl2 transgene decreased PC frequency but increased MemB abundance, with a significant interaction between the genotypes indicating a greater than additive effect (Fig. 2 G). These data reveal that in the context of PerC MemB accumulation, GPR34 KI provides signals that are non-redundant with Bcl2. Furthermore, there exists at least one condition in which KI PC and MemB can be regulated in opposing directions.

### GPR34 KI enhances MemB proliferation

**Figure 3.**
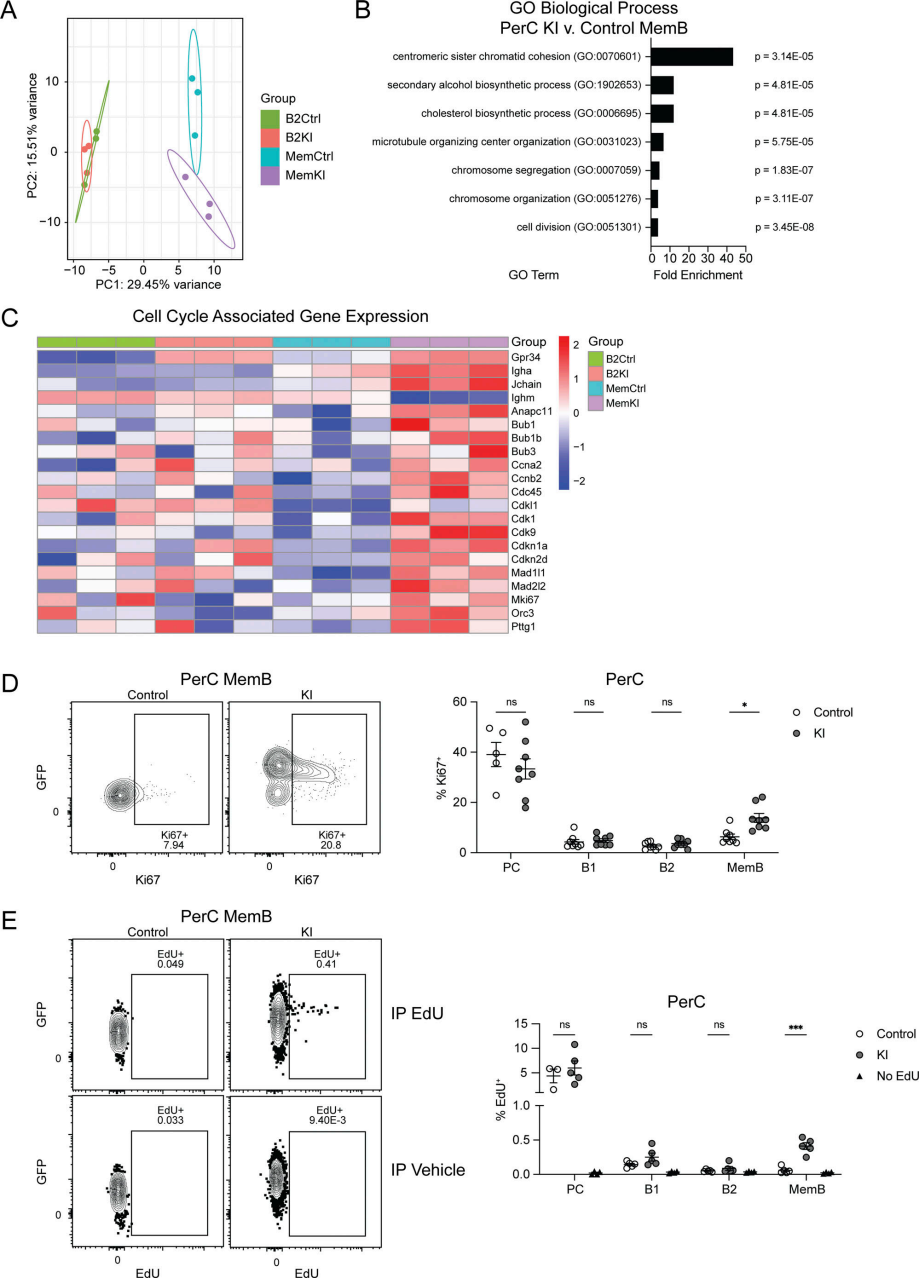
**GPR34 KI promotes MemB proliferation. (A–C)** B2 (IgD^+^IgM^+^) and MemB (IgD^−^IgM^−^) cells were sorted from the PerC from control and KI (*Cd21*^*Cre*^*R26*^*LSL-Gpr34Q340X-IRES-GFP*^) mice for bulk RNAseq (control B2, *n* = 3; control MemB, *n* = 3; KI B2, *n* = 3; KI MemB, *n* = 3). **(A)** PCA plot of sequencing samples. **(B)** GO analysis of top-upregulated DEGs comparing KI MemB to control MemB. Bar graph shows the seven GO terms with the highest fold enrichment along with each term’s P value. **(C)** Heatmap comparing normalized expression of *Gpr34*, *Igha*, *Jchain*, *Ighm*, and selected cell cycle–associated genes across the indicated populations. **(D and E)** Immune cells from control and KI mice were analyzed by flow cytometry. **(D)** Left: Representative flow cytometry plots of PerC MemB gated for Ki67^+^ cells and annotated with Ki67^+^ frequency. Right: Percentages of Ki67^+^ cells within PerC PC (CD138^+^), B1 (IgD^+^IgM^+^), B2, and MemB (control PC, *n* = 5; other control, *n* = 8; KI, *n* = 8). **(E)** Left: Representative flow cytometry plots of PerC MemB gated for EdU^+^ cells and annotated with EdU^+^ frequency. Right: Percentages of EdU incorporation in the indicated PerC populations 90 min after IP injection of EdU or vehicle (control PC, *n* = 3; other control, *n* = 5; KI, *n* = 5; no EdU, *n* = 4). Each data point represents an individual mouse (D and E), lines indicate means, and error bars represent SEM. Data were pooled from three (D) or two (E) independent experiments. Statistical significance (D and E) was determined by unpaired *t* test corrected for multiple comparisons (Holm-Šídák). ns, not significant; *P < 0.05; ***P < 0.001.

**Figure S3.**
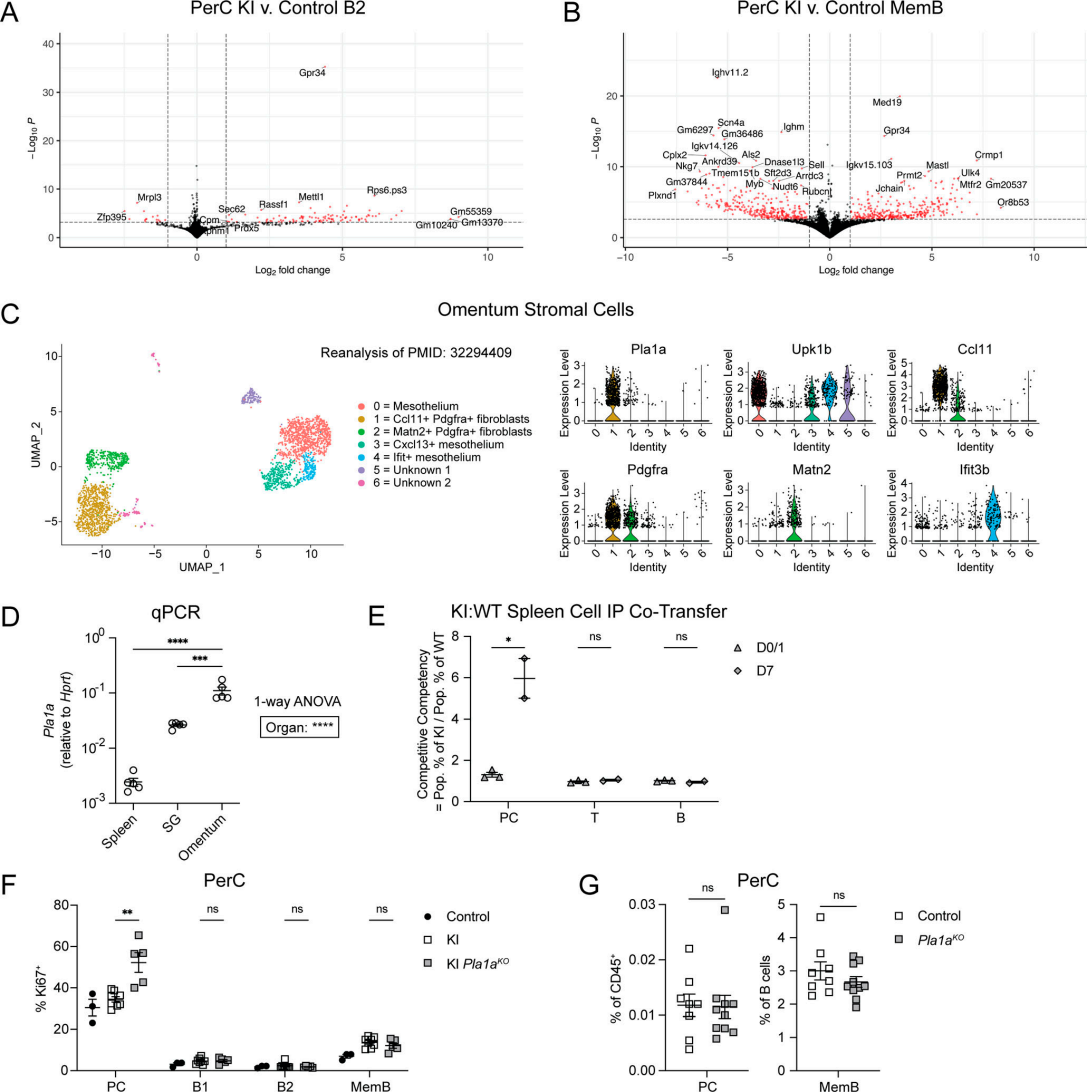
**GPR34 KI alters gene expression of PerC cells, omental fibroblasts express PLA1A, and GPR34 KI supports PC maintenance in the PerC. (A and B)** B2 (IgD^+^IgM^+^) and MemB (IgD^−^IgM^−^) cells were sorted from the PerC from control and KI mice for bulk RNAseq (control B2, *n* = 3; control MemB, *n* = 3; KI B2, *n* = 3; KI MemB, *n* = 3). Volcano plots display in red the DEGs with a log_2_ fold change >1 and an adjusted P < 0.05 for the indicated comparisons. **(C)** Seurat reanalysis of the mouse omental stromal cell scRNAseq dataset from Jackson-Jones et al. (2020). Violin plots show normalized expression of *Pla1a* and cluster-defining markers. **(D)** qPCR for expression of *Pla1a* (relative to *Hprt*) in whole spleen, SG, and omentum tissues from WT mice (spleen, *n* = 5; SG, *n* = 5; omentum, *n* = 5). **(E–G)** Immune cells were analyzed by flow cytometry. **(E)** Spleen cells from control and KI (*Cd21*^*Cre*^*R26*^*LSL-Gpr34Q340X-IRES-GFP*^) mice were harvested, dye-labeled, mixed, and co-transferred IP into congenic recipients. Donor cell recovery from recipients’ PerC was analyzed by flow cytometry for PC (CD138^+^), T cells (TCRb^+^), and B cells (CD19^+^) on the indicated days after transfer. For a given population, the competitive competency is defined as the ratio between its frequency among KI cells and its frequency among control cells, which reflects the multiplicity of the numerical advantage of KI over control. **(F)** Frequencies of Ki67^+^ cells within the indicated PerC populations from control, KI, and KI *Pla1a*^*KO*^ mice (control, *n* = 4; KI, *n* = 9; KI *Pla1a*^*KO*^, *n* = 5). **(G)** Frequencies of the indicated PerC populations in control (*Pla1a*^*WT*^ or ^*Het*^) and *Pla1a*^*KO*^ mice (control, *n* = 8; *Pla1a*^*KO*^, *n* = 10). Each data point represents an individual mouse (D–G), lines indicate means, and error bars represent SEM. In D–G, data were pooled from two independent experiments, except in E, which was from one experiment. Related to E, similar findings were obtained in separate groups of recipient mice that received donor mLN and PP cells. Statistical significance was determined by one-way ANOVA, followed by paired *t* test of each tissue to the omentum corrected for multiple comparisons (Holm-Šídák) (D) or by unpaired *t* test corrected for multiple comparisons (Holm-Šídák) (E–G). ns, not significant; *P < 0.05; **P < 0.01; ***P < 0.001; ****P < 0.0001.

Given previous reports of GPR34’s pro-growth properties in vitro (Ansell et al., 2012; Jin et al., 2015), we wondered if PerC KI cells have increased cell proliferation. PerC B2 and MemB were sorted from control and KI mice for bulk RNA sequencing (RNAseq). Principal component analysis (PCA) revealed prominent transcriptional differences between B2 and MemB along PC1; control and KI B2 were relatively similar, while PC2 captured differences between control and KI MemB (Fig. 3 A). Consistently, there were a much greater number of differentially expressed genes (DEGs) when comparing KI versus control MemB than when comparing KI versus control B2 (Fig. S3, A and B; and Table S1). Gene Ontology (GO) analysis of genes upregulated in KI MemB revealed enrichment for terms associated with “cell division” (Fig. 3 B). Accordingly, many cell cycle–related transcripts, including *Cdc45*, *Cdk1*, *Cdkn1a*, and *Mki67* were upregulated (Fig. 3 C). Intracellular staining confirmed an elevated frequency of Ki67^+^ MemB in KI compared with control (Fig. 3 D). KI PC, B1, and B2 did not exhibit this increase, although PC Ki67 levels were generally high (Fig. 3 D). As an orthogonal measurement of cell proliferation, we administered a single IP pulse of 5-ethynyl-2′-deoxyuridine (EdU) 90 min before analysis. KI MemB incorporated more EdU than their control counterparts, with the PC, B1, and B2 populations largely unaffected (Fig. 3 E).

### The omentum is enriched for KI PC and MemB

**Figure 4.**
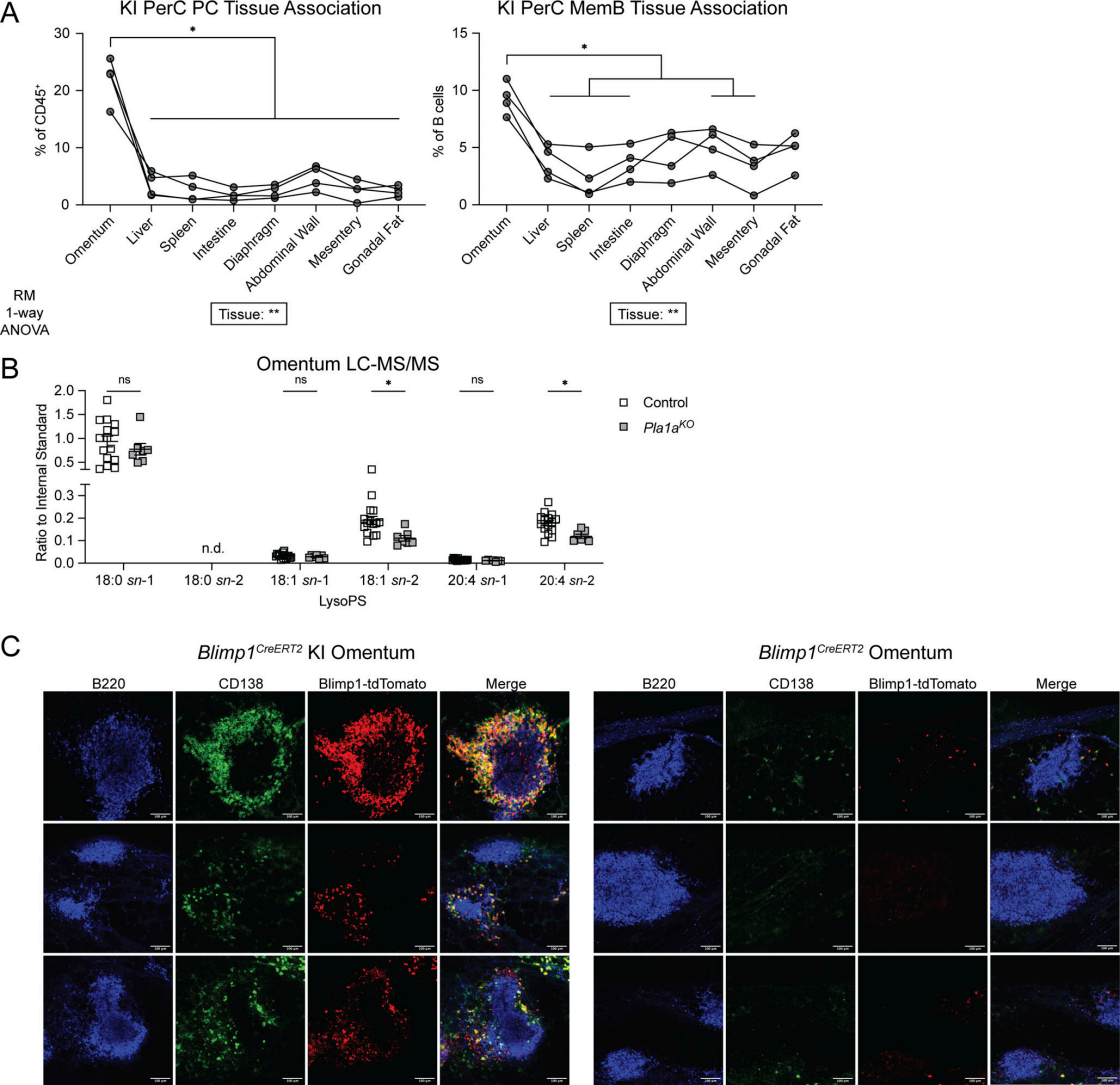
**GPR34 KI PC and MemB are enriched at the omentum where PLA1A regulates lysoPS abundance. (A)** PerC organs from KI (*Cd21*^*Cre*^*R26*^*LSL-Gpr34Q340X-IRES-GFP*^) mice or KI BM chimeric mice were harvested and rinsed in MACS buffer to dissociate cells physically associated with the visceral peritoneal surface of each tissue. Plots show frequencies of PC (CD138^+^) and MemB (IgD^−^IgM^−^) in these tissue rinses analyzed by flow cytometry. Each set of connected points represents data from one mouse (*n* = 4). Data are pooled from two independent experiments. Statistical significance was determined by repeated measures one-way ANOVA with the Geisser-Greenhouse correction, followed by paired *t* test of each tissue to the omentum corrected for multiple comparisons (Holm-Šídák). **(B)** LC-MS/MS quantification of the indicated *sn*-1 and *sn*-2 lysoPS species in the homogenized omentum of control (*Pla1a*^*WT*^ or ^*Het*^) and *Pla1a*^*KO*^ mice (control, *n* = 15; *Pla1a*^*KO*^, *n* = 7). Data were normalized to an internal standard. Each data point represents an individual mouse, lines indicate means, and error bars represent SEM. Data are pooled from two independent experiments. Statistical significance was determined by unpaired *t* test corrected for multiple comparisons (Holm-Šídák). **(C)** Representative immunofluorescence images of omenta from *Blimp1*^*CreERT2*^ KI (*Blimp1*^*CreERT2*^*R26*^*LSL-GPR34Q340X-IRES-GFP*^) (*n* = 3) and *Blimp1*^*CreERT2*^ control mice (*n* = 2) 4 days after tamoxifen treatment. PC are CD138^+^Blimp1-tdTomato^+^. Images are focused on individual FALCs, and three examples from each strain are shown. Images were pooled from three independent experiments. Scale bar, 100 µm. n.d., not detected; ns, not significant; *P < 0.05.

We next asked whether KI PerC cells were equivalently distributed across the surfaces of PerC organs. Rinsing off surface-associated cells from various tissues revealed a striking enrichment of KI PC and MemB at the omentum (Fig. 4 A). The omentum is a hub for local PerC immunity (Rangel-Moreno et al., 2009; Perez-Shibayama et al., 2018), and previous studies have shown that omental stroma expresses chemokines to recruit immune cells (Ansel et al., 2002; Jackson-Jones et al., 2020). While the rinsing data do not demonstrate that the KI cells entered the PerC through the omentum, they are consistent with this possibility. To explore whether the GPR34 ligand lysoPS is likely to be produced at the omentum, we reanalyzed a published single-cell RNAseq (scRNAseq) dataset of omental stromal cells (Jackson-Jones et al., 2020) and found that transcripts of the lysoPS-generating enzyme PLA1A were well-expressed in the *Ccl11*^*+*^*Pdgfra*^*+*^ fibroblast subset (Fig. S3 C). On the whole-tissue level, *Pla1a* transcripts were significantly more abundant in the omentum than in the SG and spleen (Fig. S3 D). Liquid chromatography–tandem mass spectrometry (LC-MS/MS) quantification of lysoPS revealed that omentum tissue from *Pla1a*^*KO*^ mice contained a lower abundance of *sn*-2 18:1 and 20:4 while the *sn*-1 forms were unchanged (Fig. 4 B). The remaining *sn*-2 lysoPS detected in PLA1A-deficient mice may be an artifact of sample preparation or may truly reflect in vivo levels, although it is unclear if there exist additional *sn*-2 lysoPS-generating enzymes (Omi et al., 2021). In our efforts to reliably identify KI PC on the omentum by immunofluorescence whole-mount microscopy, we were greatly aided by the Blimp1-tdTomato reporter. In Blimp1Cre KI omenta, CD138^+^Blimp1-tdTomato^+^ KI PC were mostly observed surrounding B220^+^ FALCs, and PC were rarely detected in control omenta (Fig. 4 C).

### Stromal PLA1A is required for PerC KI PC and MemB maintenance

**Figure 5.**
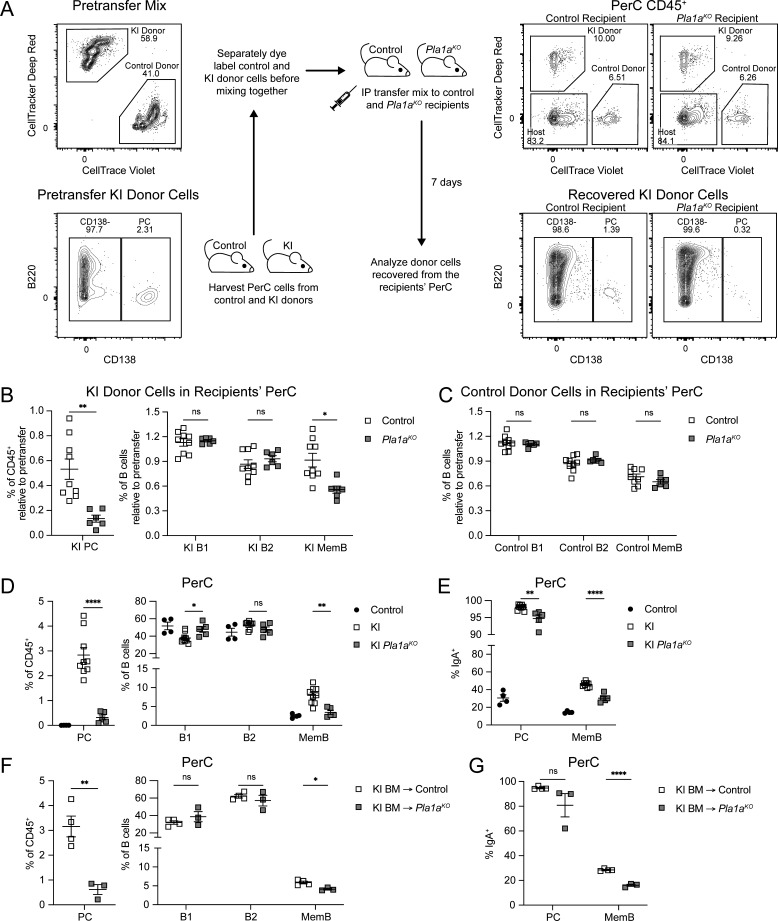
**GPR34 KI PerC PC and MemB maintenance depends on stromal PLA1A. (A–G)** PerC immune cells were analyzed by flow cytometry. **(A–C)** PerC cells from control and KI (*Cd21*^*Cre*^*R26*^*LSL-Gpr34Q340X-IRES-GFP*^) mice were dye-labeled, mixed, and IP transferred into control (*Pla1a*^*WT*^ or ^*Het*^) or *Pla1a*^*KO*^ recipient mice, whose PerC cells were analyzed on day 7. **(A)** Left top: Representative flow cytometry plot of pretransfer mix gated for dye-labeled donor cells. Left bottom: Representative flow cytometry plot of pretransfer KI donor cells gated for PC (CD138^+^) and annotated with PC frequency. Middle: Schematic of IP adoptive transfer experiment. Right top: Representative flow cytometry plots of recipient PerC CD45^+^ cells gated for donor cells. Right bottom: Representative flow cytometry plots of recovered KI donor cells gated for PC and annotated with PC frequency. **(B and C)** Plots show the frequencies of recovered co-transferred PC, B1 (IgD^−^IgM^+^), B2 (IgD^+^IgM^+^), and MemB (IgD^−^IgM^−^) normalized to their respective pretransfer frequencies (control, *n* = 9; *Pla1a*^*KO*^, *n* = 6). **(D)** Frequencies of the indicated populations in control mice and in KI mice on either a control or *Pla1a*^*KO*^ background (control, *n* = 4; KI, *n* = 9; KI *Pla1a*^*KO*^, *n* = 5). **(E)** Frequencies of IgA^+^ cells within the indicated populations (control, *n* = 4; KI, *n* = 9; KI *Pla1a*^*KO*^, *n* = 5). **(F)** Frequencies of the indicated populations in chimeric mice with KI BM reconstituted into control or *Pla1a*^*KO*^ hosts (control, *n* = 4; *Pla1a*^*KO*^, *n* = 3). **(G)** Frequencies of IgA^+^ cells within the indicated populations (control, *n* = 4; *Pla1a*^*KO*^, *n* = 3). Each data point represents an individual mouse, lines indicate means, and error bars represent SEM. Data were pooled from three (B and C) or two (D–G) independent experiments. Statistical significance (B–G) was determined by unpaired *t* test corrected for multiple comparisons (Holm-Šídák). ns, not significant; *P < 0.05; **P < 0.01; ****P < 0.0001.

To test the ligand dependence of KI cell accumulation in the PerC, we co-transferred control and KI PerC cells via IP injection into PLA1A-sufficient and -deficient recipient mice (Fig. 5 A). At 7 days, there was decreased recovery of donor KI PC and MemB from *Pla1a*^*KO*^ recipients compared with controls (Fig. 5, A and B). Recovery of donor control MemB was unaffected, as was the recovery of other populations (Fig. 5 C). These results indicate that KI PC maintenance is PLA1A dependent and that KI MemB maintenance is both PLA1A dependent and GPR34 dependent. To obtain sufficient control PC for comparison to their KI counterparts, we IP co-transferred an equal mixture of control and KI spleen cells into WT mice. At 7 days, there was much greater recovery of donor KI PC than control PC, while B cells as a whole were unaffected (Fig. S3 E). Thus, KI PC maintenance in the PerC is also GPR34 dependent.

Next, we studied the PLA1A dependence of PerC KI PC and MemB by intercrossing KI and *Pla1a*^*KO*^ mice. KI mice lacking the *Pla1a* gene showed an almost complete block in PerC accumulation of KI PC and MemB (Fig. 5 D). The increased IgA positivity of KI PC and MemB was also partially dependent on PLA1A (Fig. 5 E). However, enzyme deficiency had minimal effect on the Ki67^+^ frequency of KI MemB (Fig. S3 F), and amongst the PC remaining in the PerC of these mice, the Ki67^+^ fraction was elevated. These data suggest that in contrast to the effect on cell accumulation, KI MemB proliferation in the PerC does not require a ligand generated by PLA1A. To determine the contribution of stromal PLA1A to KI cell accumulation, we made BM chimeras in which we reconstituted PLA1A-deficient or control hosts with KI BM. In the *Pla1a*^*KO*^ hosts, enzyme expression is lacking in the stromal compartment but intact in the hematopoietic system. The KI PC and MemB frequencies were reduced in *Pla1a*^*KO*^ hosts (Fig. 5 F), signifying a substantial dependence on stroma-derived lysoPS. Stromal PLA1A also contributed to the enrichment of KI IgA^+^ cells (Fig. 5 G). Non-KI PC and MemB were not affected by PLA1A deficiency (Fig. S3 G).

### Concluding remarks

Our work reveals that GPR34 can drive the ligand-dependent accumulation of immune cells in the PerC. Immune cell homing is guided by many inputs, and it is likely that GPR34 cooperates with other factors to enable preferential PerC localization. Although with a smaller effect size, the GPR34 GOF allele also promoted PC accumulation in the PleuralC. Prior in vitro studies showed that *sn*-2 lysoPS is the more potent and likely physiological form of GPR34 ligand (Kitamura et al., 2012; Uwamizu et al., 2015; Izume et al., 2024), but the in vivo relevance of PLA1A-generated lysoPS has been elusive. The serendipitous discovery of GPR34 KI-mediated PerC PC and MemB accumulation allowed us to probe this enzyme–ligand–receptor axis. Omental *sn*-2 lysoPS levels and PerC KI cell maintenance depended on PLA1A, supporting a model where stromal cells regulate lipid mediators that govern immune cell residence. Further study is needed to understand the spatial distribution of *sn*-2 lysoPS and thus its availability to GPR34-expressing cells. The substrate of PLA1A is PS, and the relevant source of PS in the omentum at homeostasis remains unclear. Since PLA1A can generate lysoPS from PS that is externalized to the outer leaflet of dying cells (Hosono et al., 2001), we speculate that a homeostatic level of cell death may provide sufficient substrate. Alternatively, cell death may not be required at all as viable cells can also externalize PS (Zhao et al., 2021; Segawa et al., 2011; Elliott et al., 2005).

At the receptor level, GPR34 KI can promote cell cycle activation as well as ex vivo migration of B-lineage lymphocytes, but further investigation will be needed to establish whether GPR34 mediates migration in vivo. We speculate that the PerC accumulation of KI PC and MemB involves cell recruitment from circulation or possibly through lymphatics or even across the visceral peritoneal linings of PerC organs. Cell proliferation within the PerC then further contributes to PC and MemB abundance. It is also possible that some PerC PC are derived from local MemB rather than traveling from the PP. The proposed cell recruitment step likely occurs at a low rate, making it problematic to detect using conventional adoptive transfer approaches and requiring the generation of new methods for its study. Although we favor a role for GPR34 in promoting migration to the PerC, our data are also consistent with the receptor promoting retention of cells already inhabiting the PerC. Our finding that the pro-proliferative activity of GPR34 in PerC MemB was not dependent on PLA1A may be explained by the GOF receptor having sufficient constitutive activity to engage proliferative pathways in these cells or by the existence of PLA1A-independent ligands (such as *sn*-1 forms of lysoPS) in the PerC.

Although we did not observe SG KI cell accumulation or lymphoma in our mouse model (even in mice aged over 6 mo, unpublished data), we hypothesize that GPR34 GOF-driven proliferation and migration contribute to the eventual development of SG MALT lymphomas in a Sjogren’s syndrome context (Korona et al., 2022; Pringle et al., 2022). Autoimmune inflammation could potentially boost PLA1A expression in the SG, and cell death in lymphoepithelial lesions would increase the availability of PS substrate that can be converted to lysoPS. In addition, we speculate that GPR34 cooperates with other factors in promoting KI cell localization in the PerC and that those cooperating factors may be lacking in the uninflamed SG. Intriguingly, PC have been observed in up to one-third of SG MALT lymphomas (Molina et al., 2011) as well as in MALT lymphomas more generally, with occasional cases where PC constitute >80% of an extranodal tumor’s total cellularity (Wöhrer et al., 2007). The expansion of PerC PC in our model also echoes rare PerC plasmacytomas in patients (Thambi et al., 2018), including cases specifically associated with the omentum (Peison et al., 1980; Soliman et al., 2019). It remains unknown whether GPR34 plays a role in the PerC localization of PC malignancy. Finally, although we did not identify an effect of GPR34 deficiency on MemB or PC abundance in the PerC in our immunization studies, it remains possible that there exist physiologic conditions where they home to or accumulate in the PerC in a GPR34-dependent manner. Overall, this work connects PLA1A and GPR34 biology to define a ligand–receptor system that has the capacity to promote cell compartmentalization in the PerC.

## Materials and methods

### Mice

*R26*^*LSL-Gpr34Q340X-IRES-GFP*^ mice were generated on a C56BL/6N background by CRISPR/Cas9-mediated targeting of and homologous recombination at the *Rosa26* locus (Biocytogen Boston Corp.), resulting in the conditional KI allele shown in Fig. 1 A. *Pla1a*^*KO*^ (*Pla1a*^*tm1a(EUCOMM)Wtsi*^) mice were generated from ES cells of a C56BL/6N background purchased from EUCOMM. *Cd21*^*Cre*^ (Kraus et al., 2004), *Gpr34*^*KO*^ (Liebscher et al., 2011), *Blimp1*^*CreERT2*^ (Robinson et al., 2023), and E*µ-bcl2* (Strasser et al., 1991) mice have been described previously. *Blimp1*^*CreERT2*^ mice were provided by D. Tarlinton (Monash University, Melbourne, Australia). Mice were analyzed at 8–20 wk of age. Congenic hosts for BM chimeras and recipients for transfer experiments were either B6 CD45.1 (002014) mice bred internally from founders ordered from The Jackson Laboratory or B6-Ly5.1/Cr (564) mice purchased from the National Cancer Institute at Charles River at age 6–8 wk. To produce BM chimeric mice, hosts were lethally irradiated with 900 cGy X-ray irradiation (split dose separated by 3 h), followed by IV injection of BM cells from donors. Chimeras were analyzed for donor-derived immune cells 8–12 wk after reconstitution. Mice were co-caged with littermates for all experiments. All data are representative of male and female mice. Control and experimental treatments were administered to age- and sex-matched mice that had been allocated to groups randomly, with sample sizes chosen based on previous experience. The investigators were not blinded. Animals were housed in a specific pathogen-free environment in the Laboratory Animal Research Center at the University of California, San Francisco (UCSF), and all experiments conformed to ethical principles and guidelines approved by the UCSF Institutional Animal Care and Use Committee.

### Treatments and immunizations

For conditional activation of *Blimp1*^*CreERT2*^, 6 mg tamoxifen (Sigma-Aldrich) was administered via oral gavage on days 0 and 1 (Robinson et al., 2023). Mice were analyzed at various timepoints thereafter. For labeling of proliferating cells, mice were IP-injected with 0.5 mg of EdU (Thermo Fisher Scientific) 90 min before analysis. To induce a PerC PC response, mice were immunized IP with 100 μg NP30-KLH (Biosearch Technologies) in 2% Alhydrogel alum adjuvant (Invivogen).

### Cell preparation

Peritoneal and pleural lavage cells were isolated by flushing the PerC and PleuralC with 5 or 1 ml of MACS buffer (PBS containing 2% newborn calf serum [NBCS] and 1 mM EDTA), respectively. To collect cells physically associated with the visceral peritoneal surface of tissues, mice were transcardially perfused with PBS to reduce blood contamination before the PerC was opened. PerC organs were harvested and rinsed in 5 ml MACS, and the MACS rinse media was analyzed. Cell suspensions were prepared from the spleen, iLN, mLN, PP, and cLN by gentle mashing through a 70-μm strainer. The liver was mashed through a 100-µm strainer and purified for immune cells by resuspending in 40% Percoll (Thermo Fisher Scientific) and centrifuging at 2,500 rpm for 20 min at room temperature on low brake. The immune cell pellet was collected from the bottom of the tube. BM cells from leg bones were extracted by centrifugation in a microfuge at 10,000 rpm for 3 min at 4°C. To generate SG immune cell suspensions, the submandibular and sublingual glands were dissected and minced using scissors. The minced SG tissue was then incubated for 30 min shaking (1,000 rpm) at 37°C in 1 ml of RPMI with 2% NBCS, 10 mM HEPES, 1 mg/ml collagenase type IV (Worthington), and 20 µg/ml DNase I (Sigma-Aldrich). To stop the digestion, 20 μl of 500 mM EDTA was added to each sample. The digested SG tissue was mashed through a 100-μm strainer, washed with MACS buffer, and purified for immune cells using 40% Percoll. To prepare SI LP samples, PP and intestinal contents were removed from the SI, and the first 12 cm (duodenum) were cut into several pieces for further processing. The SI tissue was twice incubated for 20 min shaking (250 rpm) at 37°C in 12 ml of intraepithelial lymphocyte media (HBSS containing 5% FBS, 2 mM EDTA, and 1 mM dithiothreitol [Sigma-Aldrich]). The intraepithelial lymphocyte fractions were discarded. The tissue was then twice digested for 20 min in 12 ml of LP media (RPMI containing 10% FBS, 100 µg/ml DNase I, and 0.232 mg/ml collagenase VIII from *Clostridium histolyticum* [Sigma-Aldrich]). The two LP fractions were aggregated and purified using 40% Percoll.

### Adoptive transfer experiments

For IV transfers, PerC, spleen, mLN, and PP cell suspensions were prepared from congenically marked control and KI donor mice. These cells were transferred IV via retro-orbital injection into congenically marked recipient mice, whose PerC and spleen were analyzed after 3 days for quantification of donor cells.

For IP transfers, donor control and KI PerC cells were dye-labeled with CellTrace Violet (Invitrogen) and CellTracker Deep Red (Invitrogen), respectively. These cells were then co-transferred via IP injection into control or *Pla1a*^*KO*^ mice. In one experiment, donor control and KI spleen cells were similarly dye-labeled and co-transferred IP into congenic WT mice. Recipients’ PerC were analyzed for donor cell maintenance after 7 days.

### Flow cytometry

Cells were washed, blocked with 2.4G2 antibody (Bio X Cell), and stained for 30 min on ice in MACS buffer. The following antibodies were used: CD11b-BUV496 (BD), CD19-BUV563 (BD), IgM-BUV661 (BD), TCRb-BUV737 (BD), CD138-BV421 (Biolegend), CD45.2-BV421 (Biolegend), CD21/35-PB (Biolegend), GL7-PB (Biolegend), B220-BV510 (Biolegend), CD11b-BV570 (Biolegend), CD19-BV605 (Biolegend), CD45.1-BV605 (Biolegend), CD45.2-BV605 (Biolegend), IgD-BV650 (Biolegend), CD45.1-BV650 (Biolegend), CD138-BV711 (Biolegend), IgD-BV711 (Biolegend), CD98-BV711 (BD), EpCAM-BV711 (Biolegend), CD95-BV750 (BD), CD11b-BV785 (Biolegend), CD138-BV785 (Biolegend), CD45.1-BV785 (Biolegend), CD95-BV786 (BD), CD45-AF532 (Invitrogen), CD21/35-PE (BD), CD23-PE (BD), GL7-PE (eBioscience), IgA-PE (Southern Biotech), CD45.2-PE (Biolegend), CD23-PE/Cy7 (Biolegend), CD38-PE/Cy7 (Biolegend), IgM-PE/Cy7 (eBioscience), CD45.1-PE/Cy7 (eBioscience), IgM-PerCP/Cy5.5 (Biolegend), GL7-PerCP/Cy5.5 (Biolegend), CD45.2-PerCP/Cy5.5 (Tonbo), CD38-BB700 (BD), CCR9-APC (eBioscience), CD45.1-APC (Cytek), CD21/35-AF647 (Biolegend), CD38-AF647 (Biolegend), CD45.2-AF647 (Biolegend), GL7-AF647 (Biolegend), B220-AF700 (Biolegend), CD38-AF700 (eBioscience), CD45.1-AF700 (Biolegend), CD45.2-AF700 (Biolegend), CD4-APC/Cy7 (Cytek), CD8a-APC/Cy7 (Cytek), Ly6G-APC/Cy7 (Cytek), F4/80-APC/Cy7 (Biolegend), B220-APC/Fire810 (Biolegend), and IgA-biotin (Southern Biotech) (followed by streptavidin-BV605 [Biolegend] or streptavidin-BV711 [BD]). Dead cells were excluded using Fixable Viability Dye eFluor780 (eBioscience).

For Ki67 detection, cells were stained with surface antibodies, pre-fixed with 1% paraformaldehyde in PBS for 10 min on ice, and fixed and permeabilized with the Foxp3/Transcription Factor Staining Buffer Set (eBioscience), before intracellular staining with the Ki67-AF647 antibody (BD). For detection of EdU incorporation, the Click-iT Plus EdU Flow Cytometry Assay Kit (Invitrogen) was used. GPR34 surface staining was enabled by a polyclonal rabbit antibody raised against the first 40 N-terminal amino acids of mouse GPR34 (Biomatik). The polyclonal reagent was cross-absorbed on a *Gpr34*^*KO*^ spleen cell suspension overnight to reduce non-specific binding. After blocking, WT and KI cells were stained with the polyclonal rabbit anti-GPR34 antibody for 1 h at room temperature, followed by donkey anti-rabbit biotin-SP (Jackson Immunoresearch) for 20 min on ice, and finally SA-AF647 (Invitrogen) and other surface markers for 30 min on ice.

All samples were run on a Cytek Aurora. Flow cytometry data were analyzed using FlowJo (v10.10.0).

### Cell sorting and RNAseq

B2 (IgD^+^IgM^+^) and MemB (IgD^−^IgM^−^) cells were sorted from the PerC of control and KI mice using a BD FACSAria II. Sort purity was ∼95%. The sequencing library was prepared using Ovation RNA-seq System V2 from Nugen, the KAPA Hyper prep labeling kit, and the NEXTflex DNA barcodes Adapter kit from Bioo Scientific. 50 bp paired-end reads were acquired on a NovaSeq X at the UCSF Center for Advanced Technology. FastQC was used to examine the quality of raw sequencing reads, and cutadapt was used to trim adaptors and clean reads. Sequences were aligned to the mm39 genome with STAR, followed by quantification with RSEM, PCA with pcaExplorer, and differential gene expression analysis with DEseq2. Cutoffs for DEGs were set at a log_2_ fold change >1 and an adjusted P < 0.05. DEGs between KI and control were subjected to GO analysis (https://geneontology.org/). The PCA plot was generated by pcaExplorer. The heatmap was generated with pheatmap. Volcano plots were generated with EnhancedVolcano.

### Published scRNAseq data analysis

The Seurat R package (https://satijalab.org/seurat/) was used to reanalyze an omental stromal cell scRNAseq dataset (Jackson-Jones et al., 2020).

### Generation of GPR34-expressing WEHI-231 cells

Mouse *Gpr34* or *Gpr34R337X* was cloned into the pQEF retroviral vector (Yang and Allen, 2018) followed by a P2A-Thy1.1 as an expression marker. Retrovirus was generated by transfecting the Plat-E packaging cell line with 1.5 µg plasmid DNA and 3 μl Lipofectamine 2000 (Life Technologies). WEHI-231 mouse B lymphoma cells were grown in RPMI containing 10% FBS, 10 mM HEPES, 2 mM glutamine, 55 μM 2-mercaptoethanol, and 50 U penicillin/streptomycin. 5 × 10^5^ WEHI-231 cells were placed in a well of a 6-well plate along with the retroviral supernatant. The cells were centrifuged at 2,400 rpm for 2 h at 32°C. This spinfection was repeated with fresh retrovirus 24 h later. After 1 wk, Thy1.1-expressing cells were sorted using a BD FACSAria II.

### Transwell migration assays

WEHI-231 cells were taken from T25 flask cultures and washed twice in prewarmed migration media (RPMI containing 0.5% fatty acid-free BSA, 10 mM HEPES, and 50 U penicillin/streptomycin). PerC cells, obtained from peritoneal lavage using migration media, were washed once. Both WEHI-231 and PerC cells were resuspended at 1 × 10^7^ cells per ml and resensitized for 10 min in a 37°C water bath. 100 μl of cells (1 × 10^6^ cells) were added on top of transwells (5-µm pore, Corning) with CXCL13 (Thermo Fisher Scientific), CCL25 (Biolegend), or lysoPS 18:1 (Avanti Polar Lipids) in migration media (600 μl) in the bottom chamber. The cells were allowed to migrate for 3 h, after which the cells in the bottom well were analyzed and counted by flow cytometry.

### Immunofluorescence staining and microscopy

Mice were IP-injected with 1 µg each of CD138-BV421 (Biolegend) and B220-AF647 (Biolegend) 30 min before analysis. Omenta were carefully harvested, gently dipped once in PBS, and whole-mounted fresh on Superfrost Plus microscope slides (Thermo Fisher Scientific). Images were immediately acquired with HP PL FLUOTAR 10X/0.2 AIR objective on a Stellaris DIVE confocal microscope (Leica) using LAS X software (Leica) and subsequently processed using ImageJ (v2.14.0).

### LC-MS/MS

To distinguish and quantify the *sn*-1 and *sn*-2 forms of lysoPS, we adapted an established LC-MS/MS protocol (Okudaira et al., 2014). Omenta were harvested, weighed, and immediately added to Precellys 0.5 ml soft tissue homogenizing tubes (Cayman Chemicals) containing 9 vol (relative to omentum weight) of ice-cold acidic methanol (pH 4.0) with 50 nM lysoPS 17:1 (Avanti Polar Lipids) internal standard. Samples were homogenized using a Precellys 24 homogenizer with a Cryolys cooling unit (Bertin Technologies). The homogenization program consisted of three cycles for 20 s at 6,400 rpm (with 30 s breaks) at a temperature <4°C. Lysate was recovered, the homogenizing tube was washed with 10 vol (relative to omentum weight) of acidic methanol, and the wash was combined with the lysate before centrifuging at 21,000 rpm for 10 min at 4°C. 50 μl of supernatant was concentrated by drying in a DNA Speed Vac for 20 min. Samples were resuspended in 15 μl acidic methanol and centrifuged at 21,000 rpm for 10 min at 4°C. 10 μl supernatant was transferred to glass vials with 9 mm autosampler inserts.

Samples were analyzed using a SCIEX ExionLC UPLC in series with a SCIEX QTRAP 7500. 5 μl of each sample was injected and separated using a 5 µm CAPCELL PAK C18 column (150 × 1.5 mm; Shiseido). The mobile phase scheme consisted of A (5 mM ammonium formate in H_2_O, pH 4.0) and B (5 mM ammonium formate in 95% [vol/vol] acetonitrile, pH 4.0) delivered at a flow rate of 0.250 ml/min. Analytes were separated using the following gradient: 55% B (initial), 55% B (10 min), 85% B (30 min), 85% B (37 min), 55% B (37.1 min), and 55% B (45 min). Data were acquired in negative mode using the transitions (*m/z*) 524.3 → 437.3, 522.3 → 435.3, 544.3 → 457.2, and 508.3 → 421.3 for 18:0 lysoPS, 18:1 lysoPS, 20:4 lysoPS, and 17:1 lysoPS (as an internal standard), respectively. The ion source temperature was maintained at 350°C. The spraying needle voltage was at −2 kV. Gas 1, gas 2, curtain gas, and collision gas were set at 60, 40, 40, and 9, respectively. The entrance potential was set at −10 V, and the collision exit potential was −15 V. Collision energy was −22 eV for all compounds. Peak areas were determined using SCIEX OS. Analyte abundance was quantified as the peak area ratio, defined as the peak area of the analyte divided by the peak area of the internal standard.

### Quantitative PCR (qPCR)

Total RNA from homogenized spleen, SG, and omentum was extracted using an RNeasy kit (Qiagen) and reverse-transcribed using M-MLV reverse transcriptase (Invitrogen). qPCR was performed using Power SYBR Green (Applied Biosystems) with an Applied Biosystems StepOnePlus instrument. Data were analyzed with the comparative Ct (2^−ΔΔCt^) method using *Hprt* as a housekeeping gene. The following primers were used: Pla1a, (F) 5′-TGG​AGT​TTT​ATT​TGA​AGG​AGA-3′ and (R) 5′-GTG​GGT​TAG​GAT​GAG​CCA​T-3′.

### Statistical analyses

Data were analyzed using unpaired or paired *t* tests corrected for multiple comparisons (Holm-Šídák), repeated measures one-way ANOVA, or two-way ANOVA as specified in the figure legends. Prism software (GraphPad v10.2.3) was used for all statistical analyses and to generate plots. Each experiment was repeated at least three times unless otherwise indicated in the figure legends. In summary graphs, points indicate individual samples, horizontal lines are means and error bars represent SEM. Levels of significance were defined as *P < 0.05, **P < 0.01, ***P < 0.001, and ****P < 0.0001.

### Online supplemental material

Fig. S1 shows the characterization of GPR34 KI mice. Fig. S2 shows the characterization of *Gpr34*^*KO*^ mice. Fig. S3 contains the analysis of RNAseq data and supporting data for GPR34 KI-mediated PC and MemB maintenance in the PerC. Table S1 lists DEGs between KI and control B2 and MemB.

## Supplementary Material

Table S1 lists DEGs between KI and control B2 and MemB.

## Data Availability

Bulk RNAseq data from this study have been deposited to Gene Expression Omnibus (GEO) (accession GSE274652). The reanalyzed scRNAseq dataset (Jackson-Jones et al., 2020) in Fig. S3 C is openly available in GEO (accession GSM4053741). All other data are available in the article itself and its supplementary materials and are also available upon request from the corresponding author.
